# Artificial Intelligence-Assisted Colonoscopy With or Without Mucosal Exposure Device for Detection of Colorectal Adenomas: A Meta-Analysis

**DOI:** 10.1055/a-2676-4144

**Published:** 2025-08-29

**Authors:** Azizullah Beran, Tarek Nayfeh, Daryl Ramai, Almaza Albakri, Nasir Saleem, Marco Spadaccini, Cesare Hassan, Alessandro Repici, John J. Guardiola, Douglas K. Rex

**Affiliations:** 1 1772 Division of Gastroenterology and Hepatology, Indiana University School of Medicine, Indianapolis, IN, USA; 2 10668 Evidence-based Practice Center, Mayo Clinic, Rochester, MN, USA; 3 6915 Department of Medicine, MedStar Union Memorial Hospital, Georgetown University, Baltimore, MD, USA; 4 24508 Division of Gastroenterology, Hepatology and Endoscopy, Brigham and Women's Hospital, Boston, Massachusetts, USA; 5 2569 Department of Medicine, Cleveland Clinic Foundation, Cleveland, OH, USA; 6 12250 Department of Biomedical Sciences, Humanitas University, Pieve Emanuele, Italy; 7 9268 Endoscopy Unit, IRCCS Humanitas Research Hospital, Rozzano, Milan, Italy

**Keywords:** Endoscopy Lower GI Tract, Polyps / adenomas / ..., Quality and logistical aspects, Quality management, CRC screening

## Abstract

**Background and study aims:**

Artificial intelligence (AI) and mucosal exposure devices like Endocuff have independently improved the adenoma detection rate (ADR) during colonoscopy. This meta-analysis evaluated the combined effect of Endocuff and AI versus AI alone on colorectal neoplasia detection rates.

**Methods:**

We searched PubMed, Embase, and Web of Science for randomized controlled trials (RCTs) comparing the impact of Endocuff+AI versus AI alone on colorectal neoplasia detection. Primary outcome was ADR; secondary outcomes included advanced adenoma detection rate (AADR), sessile serrated lesion detection rate (SSLDR), cecal intubation time, and withdrawal time. Pooled risk ratios (RRs) and mean differences (MDs) with 95% confidence intervals (CIs) were calculated using a random-effects model.

**Results:**

Three RCTs with 2404 subjects were included (n = 1198 Endocuff+AI vs. n = 1206 AI alone). ADR was significantly higher in the Endocuff+AI group than in the AI alone group (54% vs. 48%, respectively) (RR 1.12, 95% CI 1.03–1.21, *P* = 0.01, I ^2^ = 0%). There was a trend toward higher AADR (12.3% vs. 10%, RR 1.23, 95% CI 0.96–1.59, *P* = 0.10, I ^2^ = 17%) and SSLDR (17.6% vs. 15.5%, RR 1.16, 95% CI 0.96–1.40, *P* = 0.13, I ^2^ = 0%) in the Endocuff+AI group compared with the AI alone group, but it did not reach statistical significance. Both cecal intubation time (MD -0.61 minutes; 95% CI -1.54–0.33; *P* = 0.20; I ^2^ = 87%) and withdrawal time (MD -0.42 minutes; 95% CI -1.01–0.17; *P* = 0.17, I ^2^ = 60%) were similar between the two groups.

**Conclusions:**

Endocuff combined with AI was superior to AI alone in improving the adenoma detection rate without increasing intubation or withdrawal times.

## Introduction

Colorectal cancer (CRC) is the third most common cancer in the United States and the second leading cause of cancer-related deaths 1 . Colonoscopy is the gold standard for CRC screening, enabling detection and removal of precancerous polyps through polypectomy, which is the cornerstone of CRC prevention 2 3 . Adenoma detection rate (ADR) is a key quality metric for screening colonoscopy, with rates varying significantly among endoscopists due to its operator-dependent nature 4 5 . Reported ADRs range from 10% to 50%, with higher ADRs associated with reduced incidence and mortality of interval CRC 5 6 7 . Missed adenomas are often attributed to challenges in recognizing subtle lesions and incomplete mucosal inspection 8 .

Artificial intelligence (AI), or computer-assisted detection (CADe), is increasingly utilized for real-time colorectal polyp detection. These systems enhance endoscopic accuracy by highlighting mucosal regions that exhibit characteristics suggestive of polyps. Several randomized controlled trials (RCTs) have demonstrated that AI improves ADR 9 10 11 and reduces adenoma miss rates 12 . However, about 10% to 20% of adenomas remain undetected even with AI, often due to polyps being obscured by colonic folds or other blind spots 12 13 . Mucosal exposure devices, such as the Endocuff (Olympus, Tokyo, Japan), have proven effective in increasing mucosal exposure during colonoscopy withdrawal, thereby improving ADR 14 15 . RCTs have shown that Endocuff is effective in increasing ADR 16 17 . In this meta-analysis, we aimed to investigate the effect of a combination of Endocuff and AI versus AI alone during colonoscopy on detection rates of colorectal neoplasia.

## Methods

We adhered to the Preferred Reporting Items for Systematic Reviews and Meta-Analyses (PRISMA) guidelines 18 in presenting this systematic review. The protocol for our review was preregistered with the International Prospective Register of Systematic Reviews (PROSPERO).

### Data sources and search strategy

We searched PubMed, Embase, and Web of Science databases from inception through November 30, 2024. The following MeSH terms were used: (“Endocuff” or “mucosal exposure device”) and (“artificial intelligence” or “computer-aided detection”) and (“colonoscopy”). The search was limited to English language, because the language filter did not seem to affect the initial search results. **Supplementary Table 1** describes the full search terms used in each database searched. Two reviewers (AB and TN) independently screened and selected the potentially included studies. Discrepancies were handled by a third reviewer (DR).

### Inclusion and exclusion criteria

Only published RCTs comparing the effect of a combination of Endocuff and AI versus AI alone on detection rates of colorectal neoplasia were eligible for inclusion. Observational studies were excluded. Conference abstracts were also excluded, because this filter did not seem to affect the initial search results.

### Data extraction

Data on baseline study and subject characteristics and outcome measures were extracted by two independent reviewers (AB and TN). Extracted study characteristics included country of origin, sample size, AI system, and reported outcomes: ADR, advanced adenoma detection rate (AADR), sessile serrated lesions detection rate (SSLDR), cecal intubation time, and withdrawal time. Extracted subject characteristics included mean age, male sex percentage, mean Boston Bowel Preparation Scale (BBPS) score, cecal intubation time, withdrawal time, and indications for colonoscopy.

### Outcomes and definitions

The primary outcome was ADR, defined as the proportion of patients with at least one detected adenoma. Secondary outcomes were AADR, SSLDR, cecal intubation time (in minutes), and withdrawal time (in minutes). Advanced adenoma was defined as an adenoma either ≥ 10 mm in size, with villous component, or high-grade dysplasia. SSLDR was defined as the proportion of patients with at least one detected SSL.

### Statistical analysis

Pooled risk ratios (RRs) with corresponding confidence intervals (CI) were calculated using the random-effects model, incorporating the Mantel-Haenszel method as a foundation. To account for anticipated between-study heterogeneity, we applied a random-effects extension, as described by van Houwelingen et al. 19 , which models 2×2 tables using non-central hypergeometric distributions. *P* < 0.05 was considered statistically significant. Heterogeneity was evaluated using the I ^2^ statistic, as outlined in the Cochrane handbook for systematic reviews, and I ^2^ value ≥ 50% was considered significant statistical heterogeneity 20 . All statistical analyses were conducted via Review Manager 5.4 and Comprehensive Meta-Analysis 3.3.

### Bias assessment

The revised Cochrane risk of bias tool was used to assess quality of the RCTs. Two reviewers (AB and DR) independently assessed each study for bias. Discrepancies were resolved by a third reviewer (JG). The Grading of Recommendations, Assessment, Development, and Evaluation (GRADE) approach was used to rate certainty of evidence (CoE) for outcomes. CoE is rated high for estimates derived from RCTs. CoE is rated down for methodological limitations of the included studies (i.e., risk of bias), imprecision (using a minimally contextualized approach, for ADR, CoE was rated down once if the CI of the absolute difference in detection rate overlapped a preset threshold of 180 per 1000 derived from Lui et al 21 . For AADR and SSLDR, CoE was rated down once if the absolute difference in detection rate overlapped a preset threshold of 25 and 39 per 1000, respectively, corresponding with a relative risk difference of 25%, indirectness, inconsistency of the results (by visual inspection of the variability of the results in the forest plots and based on an arbitrary threshold of I ^2^ . 50%), or publication bias 22 23 24 25 26 27 28 . The CoE for each outcome is summarized in **Supplementary Table 2** based on the GRADE approach.

## Results

### Study selection

**Fig. 1:**
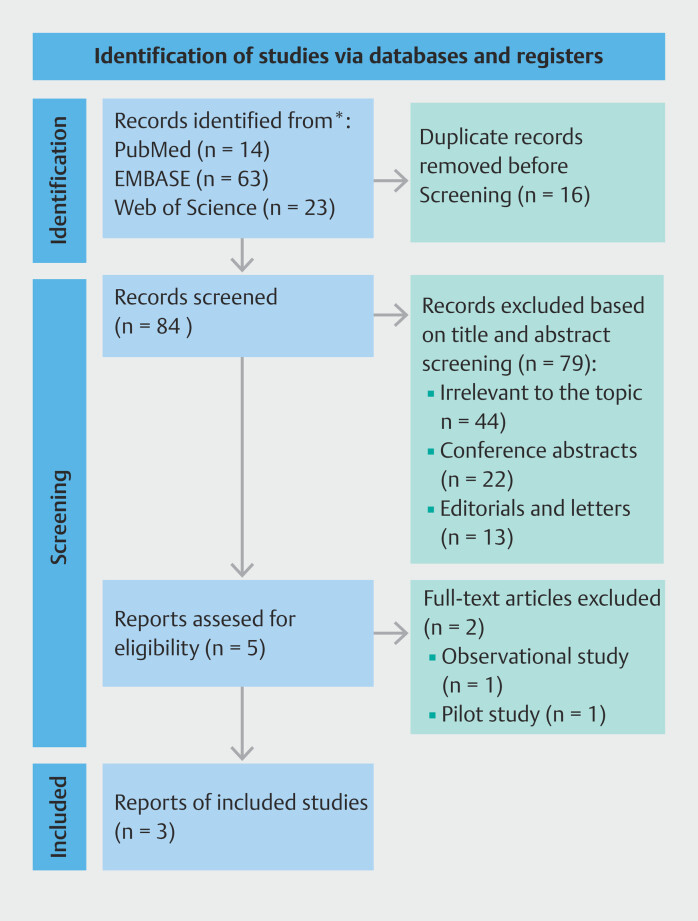
PRISMA flow diagram for selection of studies 18 .

Among the 100 studies initially screened, five met the eligibility criteria for our systematic review. However, two of these were excluded because they were observational or pilot studies. Ultimately, three studies 21 29 30 met the eligibility criteria and were included in the meta-analysis. The selection process is summarized in the PRISMA diagram in Fig. 1 .

### Study and subject characteristics

Study and subject characteristics are summarized in Table 1 . The three studies were published between March 2023 and July 2024, with one conducted in China, another in Thailand, and the third in Europe (Italy and Switzerland). A total of 2,404 subjects were included (n = 1198 Endocuff+AI vs. n = 1206 AI alone). Mean age was 62.4 ± 9.4 years, and males represented 45.9% of patients.

**Table:** **Table 1** Baseline characteristics of included studies.

	**Aniwan, 2023**		**Lui, 2024**		**Spadaccini, 2023**	
Country	Thailand		China		Multinational (Italy and Switzerland)	
AI system	Medtronic GI Genius		Olympus OIP-1		Fujifilm CAD EYE	
Total subjects, n	620		468		1316	
Comparison groups	**EC+AI**	**AI alone**	**EC+AI**	**AI alone**	**EC+AI**	**AI alone**
	308	312	230	238	660	656
Age, years, mean (SD)	61.7 (7)	62.8 (6.8)	64 (10)	66.3 (10.2)	61.5 (9.9)	61.3 (10)
Male, %	42.9%	42.6%	51.3%	55%	48.2%	47.1%
BBPS score, mean (SD)	7.9 (1.26)	8.1 (1.26)	7.3 (1.7)	6.9 (1.7)	7.14 (1.32)	7.13 (1.31)
Cecal intubation time, min, mean (SD)	5 (4–7)*	6.5 (4.1–9.4)*	6.3 (3.2)	7 (4.1)	7.37 (5.86)	7.13 (5.45)
Withdrawal time, min, mean (SD)	8.9 (5.11)	9.5 (5.56)	8.3 (5.5)	9.3 (4.6)	8.96 (2.24)	9.01 (2.48)
Indications for colonoscopy, %						
Screening	100%	100%	17.8%	15.5%	24.9%	25.4%
Surveillance	None	None	29.6%	26.5%	22.8%	22.2%
Diagnostic	None	None	52.6%	58%	32.2%	34%
AI, artificial intelligence; BBPS: Boston Bowel Preparation Scale; EC, Endocuff; SD, standard deviation. *Median (interquartile range).						

### Primary outcome: Adenoma detection rate

**Fig. 2:**
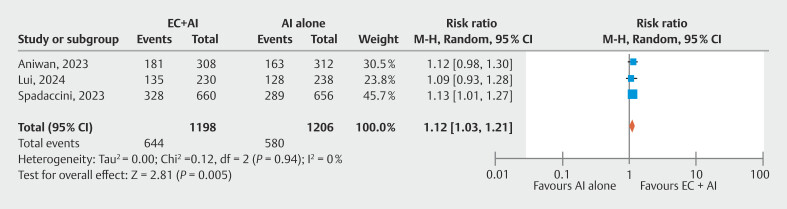
Forest plot comparing Endocuff+AI group and AI alone group for adenoma detection rate.

All three studies 21 29 30 (n = 2404) reported ADR. ADR was significantly higher in the Endocuff+AI group than in the AI alone group (54% vs. 48%, respectively) (RR 1.12, 95% CI 1.03–1.21, *P* = 0.01, I ^2^ = 0%, Fig. 2 ) (CoE was moderate, **Supplementary Table 2** ).

### Secondary outcomes: AADR, SSLDR, cecal intubation time, and withdrawal time

**Fig. 3:**
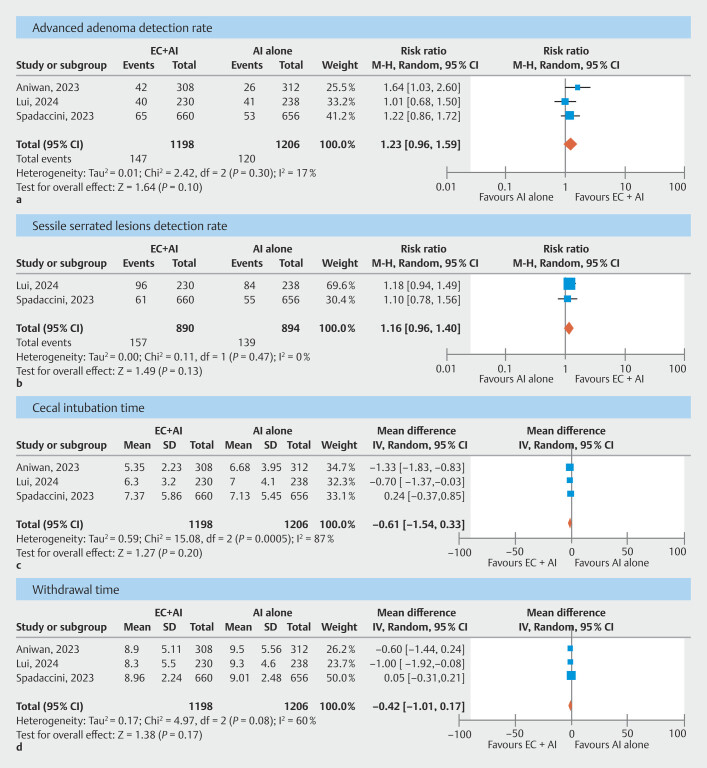
Forest plots comparing Endocuff+AI group and AI alone group for: **a** advanced adenoma detection rate, **b** sessile serrated lesion detection rate, **c** cecal intubation time, and **d** withdrawal time.

All three studies 21 29 30 (n = 2404) reported AADR and two studies 21 30 reported SSL detection rate. There was a trend toward higher AADR (12.3% vs. 10%, RR 1.23, 95% CI 0.96–1.59, *P* = 0.10, I ^2^ = 17%, moderate CoE, Fig. 3 **a** , **Supplementary Table 2** ) and SSL detection rate (17.6% vs. 15.5%, RR 1.16, 95% CI 0.96–1.40, *P* = 0.13, I ^2^ = 0%, moderate CoE, Fig. 3 **b** , **Supplementary Table 2** ) in the Endocuff+AI group compared with the AI alone group, but it did not reach statistical significance.

All three studies 21 29 30 reported cecal intubation and withdrawal time. Both cecal intubation time (6.6 ± 4.8 vs. 7 ± 4.8 minutes, MD -0.61 minutes; 95% CI -1.54 to 0.33; *P* = 0.20; I ^2^ = 87%; low CoE; Fig. 3 **c** , **Supplementary Table 2** ) and withdrawal time (8.8 ± 3.9 vs. 9.2 ± 3.9 minutes, MD -0.42 minutes; 95% CI -1.01 to 0.17; *P* = 0.17, I ^2^ = 60%, moderate CoE, Fig. 3 **d** , **Supplementary Table 2** ) were similar between the two groups.

### Bias assessment

Risk of bias was assessed and is summarized in **Supplementary Table 3** . All three included studies 21 29 30 were low risk of bias ( **Supplementary Table 3** ). Given the limited number of studies (< 10), we could not evaluate publication bias.

## Discussion

In this systematic review and meta-analysis of all RCTs investigating the combination of Endocuff with AI versus AI alone during colonoscopy, we found that combining Endocuff with AI during colonoscopy was associated with significant improvement in ADR without increasing intubation or withdrawal times compared with AI alone.

Our study revealed that combining AI with Endocuff device improved the ADR by 6% compared with AI alone. The results are consistent with the largest RCT by Spadaccini et al. 30 in Europe, which showed improvement in ADR from 44% with AI alone to 49.6% with Endocuff and AI. The European RCT used Fujifilm CAD EYE as AI system. However, the other two trials used different AI systems: Medtronic GI Genius 29 and Olympus OIP-1 21 . Despite the variation in AI systems, all three trials used the same mucosal exposure device (Olympus Endocuff Vision) and consistently showed that the combined Endocuff+AI group achieved higher ADR compared with AI alone group. This improvement is likely attributable to the ability of the Endocuff to optimize mucosal exposure by reducing blind spots caused by colonic folds, enabling AI systems to detect polyps that might otherwise be missed.

Our study observed a trend toward a higher AADR in the Endocuff+AI group compared with the AI-alone group; however, this difference did not reach statistical significance. Notably, none of the three studies were able to establish the superiority of the combined Endocuff+AI approach over AI alone in detecting advanced adenomas. This may be attributed to the relatively larger size and lower incidence rates of these advanced lesions, which could result in insufficient statistical power to detect a significant difference. Although only one RCT by Lui et al. 21 demonstrated improved detection of SSLs in the Endocuff+AI group compared with the AI-alone group, our study showed a trend toward a higher SSLDR with the Endocuff+AI group, although it was not statistically significant. A critical factor in achieving a high SSLDR is quality of bowel preparation. In the Lui et al study 21 , mean BBPS score in the Endocuff+AI group was relatively higher than in the AI-alone group (7.3 ± 1.7 vs. 6.9 ± 1.7, *P* = 0.06). Conversely, in the RCT by Spadaccini et al. 30 , both groups had comparable mean BBPS scores (7.14 vs. 7.13, *P* = 0.88). In addition, AI sometimes fails to detect flat lesions, especially large flat SSLs 31 . Moreover, only two studies were included in the analysis of SSLDR in our meta-analysis, potentially limiting the overall statistical power to detect a meaningful difference.

Although a previous RCT reported that Endocuff increased polyp detection with a shorter withdrawal time compared with high-definition colonoscopy without AI 15 , we found no significant differences in cecal intubation time (6.6 ± 4.8 vs. 7 ± 4.8 minutes, respectively) or withdrawal time (8.8 vs. 9.2 minutes, respectively) between the Endocuff+AI and AI-alone groups. Lack of a significant reduction in withdrawal time with the Endocuff+AI group compared with the AI-alone group may be explained by the increased inspection time required when using AI, partly due to the higher number of false positives flagged by the system 32 .

Adoption and utility of the Endocuff Vision device have varied across healthcare settings. Its use should be carefully individualized, because its use may be limited in patients with anatomical constraints, such as narrowed sigmoid colons due to diverticular disease, where removal of the device might be necessary. However, only a small percentage of patients (3%) require device removal 33 .

Our study has certain limitations. First, lack of blinding of endoscopists to the intervention may have introduced operator bias. Nonetheless, this limitation is common and often difficult to avoid in interventional studies. However, the blinding of pathology and outcome assessors in some of the included studies 30 may have helped mitigate this bias. Second, the trials included in this meta-analysis were conducted in only two regions (Asia and Europe), which may limit generalizability of our findings to other populations. However, the trials that previously evaluated the role of Endocuff Vision in enhancing endoscopist detection performance compared with standard colonoscopy (without AI) were conducted across multiple continents 34 . In addition, patient populations varied across studies, with different indications for colonoscopy (screening, surveillance, or diagnostic), introducing clinical heterogeneity. To account for this, we used a random-effects model. Third, all the included RCTs utilized the same mucosal exposure device, leaving it uncertain whether these findings are applicable to other mucosal exposure devices. However, our aim was not to demonstrate that mucosal exposure devices are universally effective in enhancing endoscopist performance during AI-assisted colonoscopy. Instead, our analysis—supported by the three individual studies—emphasized that AI alone may not be sufficient to address all detection challenges. Proper mucosal exposure, achieved through optimal colonoscopy techniques, remains fundamental. Notably, use of a device with well-established efficacy in improving detection performance, namely Endocuff Vision, served to reinforce this key concept 34 .

## Conclusions

In conclusion, Endocuff combined with AI was superior to AI alone in improving ADR without increasing intubation or withdrawal times. This combined approach could be an effective strategy to improve adenoma detection in colorectal cancer screening.

## Supplementary Material

Supplementary Material Zusatzmaterial
