# Supplementary material for: Artificial Intelligence-Assisted Colonoscopy With or Without Mucosal Exposure Device for Detection of Colorectal Adenomas: A Meta-Analysis
Source: Endosc Int Open. 2025 Aug 29;13:a26764144. doi: 10.1055/a-2676-4144 (PMC12417794; doi:10.1055/a-2676-4144)
Supplement: Supplementary file 1 — Supplementary Material [file 10-1055-a-2676-4144_26792559.pdf]

Supplementary Table 1 Search strategy used in each database searched.

Database	Search strategy	Articles retrieved
**PubMed**	("endocuff"[All Fields] OR (("distal"[All Fields] OR "distalization"[All Fields] OR "distalize"[All Fields] OR "distalized"[All Fields] OR "distalizer"[All Fields] OR "distalizers"[All Fields] OR "distalizes"[All Fields] OR "distalizing"[All Fields] OR "distally"[All Fields] OR "distals"[All Fields]) AND ("attach"[All Fields] OR "attachable"[All Fields] OR "attached"[All Fields] OR "attachement"[All Fields] OR "attaches"[All Fields] OR "attaching"[All Fields] OR "attachment"[All Fields] OR "attachments"[All Fields])) OR (("mucosalization"[All Fields] OR "mucosalized"[All Fields] OR "mucosally"[All Fields] OR "mucose"[All Fields] OR "mucoses"[All Fields] OR "mucositis"[MeSH Terms] OR "mucositis"[All Fields] OR "mucositides"[All Fields] OR "mucous membrane"[MeSH Terms] OR ("mucous"[All Fields] AND "membrane"[All Fields]) OR "mucous membrane"[All Fields] OR "mucosal"[All Fields]) AND ("exposure"[All Fields] OR "exposure s"[All Fields] OR "exposured"[All Fields] OR "exposures"[All Fields] OR "exposuring"[All Fields]) AND ("device s"[All Fields] OR "equipment and supplies"[MeSH Terms] OR ("equipment"[All Fields] AND "supplies"[All Fields]) OR "equipment and supplies"[All Fields] OR "device"[All Fields] OR "instrumentation"[MeSH Subheading] OR "instrumentation"[All Fields] OR "devices"[All Fields]))) AND ((("computability"[All Fields] OR "computable"[All Fields] OR "computating"[All Fields] OR "computation"[All Fields] OR "computational"[All Fields] OR "computations"[All Fields] OR "compute"[All Fields] OR "computed"[All Fields] OR "computer s"[All Fields] OR "computers"[MeSH Terms] OR "computers"[All Fields] OR "computer"[All Fields] OR "computes"[All Fields] OR "computing"[All Fields] OR "computional"[All Fields]) AND ("aided"[All Fields] OR "aiding"[All Fields]) AND ("detect"[All Fields] OR "detectabilities"[All Fields] OR "detectability"[All Fields] OR "detectable"[All Fields] OR "detectables"[All Fields] OR "detectably"[All Fields] OR "detected"[All Fields] OR "detectible"[All Fields] OR "detecting"[All Fields] OR "detection"[All Fields] OR "detections"[All Fields] OR "detects"[All Fields])) OR ("artificial intelligence"[MeSH Terms] OR ("artificial"[All Fields] AND "intelligence"[All Fields]) OR "artificial intelligence"[All Fields])) AND ("colonoscopy"[MeSH Terms] OR "colonoscopy"[All Fields] OR "colonoscopies"[All Fields])	14

**Embase**	('endocuff'/exp OR endocuff OR 'distal attachment' OR (distal AND ('attachment'/exp OR attachment)) OR 'mucosal exposure device' OR (mucosal AND ('exposure'/exp OR exposure) AND ('device'/exp OR device))) AND ('computer aided detection'/exp OR 'computer aided detection' OR (('computer'/exp OR computer) AND aided AND ('detection'/exp OR detection)) OR 'artificial intelligence'/exp OR 'artificial intelligence' OR (artificial AND ('intelligence'/exp OR intelligence))) AND ('colonoscopy'/exp OR colonoscopy)	63
**Web of Science**	(endocuff or (distal attachment) or (mucosal exposure device)) AND ((computer aided detection) OR (artificial intelligence)) AND colonoscopy	23

Supplementary Table 2 Assessment of certainty of evidence by GRADE for outcomes.

Outcomes	Number of participants (studies) Follow-up	Certainty of evidence (GRADE)	Relative effect (95% CI)	Anticipated absolute effects	
				**Risk with AI alone**	**Risk difference with Endcuff plus AI**
Adenoma Detection Rate	2404 (3 RCTs)	⊕⊕⊕⊕ High[a]	**RR 1.12** (1.03 to 1.21)	481 per 1,000	**58 more per 1,000** (14 more to 101 more)
Advanced Adenoma Detection Rate	2404 (3 RCTs)	⊕⊕⊕◯ Moderate[b]	**RR 1.23** (0.96 to 1.59)	100 per 1,000	**23 more per 1,000** (4 fewer to 59 more)
Sessile Serrated Adenoma Detection Rate	1784 (2 RCTs)	⊕⊕⊕◯ Moderate[c]	**RR 1.16** (0.96 to 1.40)	155 per 1,000	**25 more per 1,000** (6 fewer to 62 more)
Cecal Intubation Time	2404 (3 RCTs)	⊕⊕◯◯ Low[d,e]	-	The mean cecal intubation time was **6.9** minutes	MD **0.61 minutes fewer** (1.54 fewer to 0.33 more)
Withdrawal Time	2404 (3 RCTs)	⊕⊕⊕◯ Moderate[e,f]	-	The mean withdrawal time was **9.1** minutes	MD **0.42 minutes fewer** (1.01 fewer to 0.17 more)

*__Risk in the intervention group__ (and its 95% confidence interval) is based on the assumed risk in the comparison group and the **relative effect** of the intervention (and its 95% CI).
CI, confidence interval; MD, mean difference; RCT, randomized controlled trial; RR, risk ratio.

GRADE Working Group grades of evidence
High certainty: We are very confident that the true effect lies close to that of the estimate of the effect.
Moderate certainty: We are moderately confident in the effect estimate: the true effect is likely to be close to the estimate of the effect, but there is a possibility that it is substantially different.
Low certainty: Our confidence in the effect estimate is limited: the true effect may be substantially different from the estimate of the effect.
Very low certainty: We have very little confidence in the effect estimate: the true effect is likely to be substantially different from the estimate of effect.

Explanations

a. CoE not downrated for imprecision as the 95% CI of absolute risk difference does overlap the pre-set threshold of 18% based on Lui et al, 2024.
b. CoE downrated for imprecision by one level as the 95% CI of absolute risk difference overlapped the pre-set threshold of 2.5% corresponding with a relative risk difference of 25%.
c. CoE downrated for imprecision by one level as the 95% CI of absolute risk difference overlapped the pre-set threshold of 3.9% corresponding with a relative risk difference of 25%.
d. CoE downrated by two levels due to considerable heterogeneity $I^2 = 87\%$.
e. CoE not downrated for imprecision even though the 95% CI overlapped the pre-set threshold of 1 minute, as it is explained by the between-study heterogeneity.
f. CoE downrated by one level due to substantial heterogeneity $I^2 = 60\%$.

Supplementary Table 3 Quality assessment of studies in the meta-analysis.

RCTs	Random sequence generation (selection bias)	Allocation concealment (selection bias)	Blinding of participants and personal (performance bias)	Blinding of outcome assessment (detection bias)	Incomplete outcome data (attrition bias)	Selective reporting (reporting bias)
**Aniwan 2023**	+	+	?	+	+	+
**Lui 2024**	+	+	?	+	+	+
**Spadaccini 2024**	+	+	?	+	+	+

(+) low risk of bias, (−) High risk of bias, (?) Unclear risk of bias
